# Uptake and persistence of bacterial magnetite magnetosomes in a mammalian cell line: Implications for medical and biotechnological applications

**DOI:** 10.1371/journal.pone.0215657

**Published:** 2019-04-23

**Authors:** Jefferson Cypriano, Jacques Werckmann, Gabriele Vargas, Adriana Lopes dos Santos, Karen T. Silva, Pedro Leão, Fernando P. Almeida, Dennis A. Bazylinski, Marcos Farina, Ulysses Lins, Fernanda Abreu

**Affiliations:** 1 Instituto de Microbiologia Paulo de Góes, Universidade Federal do Rio de Janeiro, Rio de Janeiro, Brazil; 2 Instituto de Ciências Biomédicas, Universidade Federal do Rio de Janeiro, Rio de Janeiro, Brazil; 3 Centro Brasileiro de Pesquisas Físicas, Rio de Janeiro, Brazil; 4 Asian School of the Environment, Nanyang Technological University, Singapore, Singapore; 5 School of life Sciences, University of Nevada at Las Vegas, Las Vegas, Nevada, United States of America; Institute of Materials Science, GERMANY

## Abstract

Magnetotactic bacteria biomineralize intracellular magnetic nanocrystals surrounded by a lipid bilayer called magnetosomes. Due to their unique characteristics, magnetite magnetosomes are promising tools in Biomedicine. However, the uptake, persistence, and accumulation of magnetosomes within mammalian cells have not been well studied. Here, the endocytic pathway of magnetite magnetosomes and their effects on human cervix epithelial (HeLa) cells were studied by electron microscopy and high spatial resolution nano-analysis techniques. Transmission electron microscopy of HeLa cells after incubation with purified magnetosomes showed the presence of magnetic nanoparticles inside or outside endosomes within the cell, which suggests different modes of internalization, and that these structures persisted beyond 120 h after internalization. High-resolution transmission electron microscopy and electron energy loss spectra of internalized magnetosome crystals showed no structural or chemical changes in these structures. Although crystal morphology was preserved, iron oxide crystalline particles of approximately 5 nm near internalized magnetosomes suggests that minor degradation of the original mineral structures might occur. Cytotoxicity and microscopy analysis showed that magnetosomes did not result in any apparent effect on HeLa cells viability or morphology. Based on our results, magnetosomes have significant biocompatibility with mammalian cells and thus have great potential in medical, biotechnological applications.

## Introduction

Nano-sized magnetic particles that functionally integrate into cells and subcellular structures are versatile tools for monitoring cell position and function *in vivo*, as well as for the development of drug-delivery systems [1]. Magnetosomes biomineralized by magnetotactic bacteria (MTB) possess many attributes that make them suitable for biotechnological applications [1]. Among these attributes are the high chemical purity of the magnetic mineral, well-defined crystals morphologies, narrow size range in nanometric scale, dimensions consistent with single magnetic domains and the presence of a phospholipid bilayer surrounding each crystal. Therefore, studies involving cell-magnetosomes interactions are potentially relevant for nanobiotechnology and medicine [2].

Several applications using bacterial magnetosomes have been proposed and are currently under investigation for medical diagnosis including magnetic resonance imaging contrast, drug delivery, magnetic fluid hyperthermia therapy, and DNA extraction techniques [3–7]. Many of these applications are well described and documented, showing the advantages of using bacterial magnetosomes over synthetic magnetic nanocrystals [8]. For example, magnetosomes appear to be more effective in generating heat in magnetic fluid hyperthermia than synthetic magnetic crystals, probably due to the optimum particle size and stable magnetic properties [9]. Moreover, because of the presence of the membrane containing proteins in the structure of magnetosomes, they are more easily functionalized by bioconjugation techniques [8].

Although some studies have investigated applications involving the use of bacterial magnetosomes in medicine, few considered or examined their cytotoxicity, accumulation, and localization in mammalian cells. Liu et al. (2012) simulated lysosome activity against magnetosomes and chemically-synthesized magnetic nanoparticles using bovine pancreas proteases *in vitro* and showed that after 28 days, magnetosome crystals dissolved into free soluble iron ions [9]. When accumulation and degradation of magnetic nanoparticles were studied *in vivo* in mice, both magnetosomes and synthetic particles were observed within endocytic vesicles in liver cells 14 days after the intravenous injection of both magnetic particles in the caudal vein of the mice [9]. However, only endocytic vesicles containing magnetosomes fused to lysosomes, suggesting that magnetosomes and artificial nanoparticles underwent different decomposition pathway [9]. Other *in vivo* studies showed that the morphology of magnetosomes’ magnetite crystals was preserved after magnetosome injection in rats [10,11]. In these cases, it was suggested that magnetosomes accumulated in the liver of organisms [9,10,12] or were dissolved into free soluble iron ions or had their crystalline structure unaffected and were eliminated in feces or urine [13].

These studies are relevant and pioneer in the evaluation of the potential use of magnetosomes in humans for medical purposes. However, to date *in vitro* studies considering the cytotoxicity and the endocytic pathways of magnetosomes in human cell line models are scarce. Regardless of the medical application, magnetosomes should not lose their magnetic properties and structure during the period in which are being used as tools for drug delivery, hyperthermia or localization reporter of molecules or other structures. Therefore, we used conventional and high-resolution microscopy techniques to study the uptake and persistence of bacterial magnetosomes using HeLa cells as a model; the viability of HeLa cells after interaction with and uptake of magnetosomes; and possible modifications on the ultrastructure of the magnetosomes magnetite crystals after the internalization by HeLa cells.

## Materials and methods

### Cell cultures

HeLa cells were grown in Dulbecco Modified Eagle Medium (DMEM) with 10% fetal bovine serum (FBS) at 37°C in 5% CO_2_, as recommended by the American Type Culture Collection (ATCC). *Magnetovibrio blakemorei* strain MV-1 was cultivated anaerobically in liquid medium with nitrous oxide (N_2_O) and FeSO_4_•6H_2_O (100 μM) as the major source of Fe according to Silva et al. (2013) [14]. Cells of this strain were grown at 28°C for up to 192 h.

### Purification of magnetosomes

Cells from cultures of *M*. *blakemorei* strain MV-1 were centrifuged in 50 ml polypropylene tubes at 5,700 x g for 30 min at room temperature. The cell pellet was resuspended in 1% sodium dodecyl sulfate solution containing 0.2 M NaOH and incubated at 60°C for 15 min for cell disruption. Magnetosomes were magnetically recovered for 1 h at 4°C and the supernatant was discarded. Magnetosomes were washed in 10 mM HEPES buffer containing 200 mM NaCl (pH 7.4) in a bath sonicator (Branson 2200, Branson Ultrasonics Corporation, USA) for 15 min. The washing step was repeated 10 times. Finally, magnetosomes were resuspended in the same buffer and stored at—20°C.

### Cell titer blue cytotoxicity assay

For cytotoxicity assay, 1 x 10^4^ HeLa cells were seeded in a 96-well round bottom plates. After 12 h of incubation, an aqueous solution containing 740 μg/ml of purified magnetosomes was sonicated for 1 min. 100 μl of this suspension was added to each well followed by incubation for 24 h at 37°C in 5% CO_2_. After this period, cells were washed with phosphate buffered saline (PBS) buffer to remove magnetosomes not taken up by or attached to cells. Cytotoxic activity associated with magnetosome persistence was evaluated at 24 h intervals up to 120 h. The cytotoxic effect of magnetosomes on HeLa cells was evaluated by the CellTiter-Blue cell viability assay according to the manufacturer’s instructions (Promega, USA). Fluorescence intensity was measured by Fluoroskan Ascent fluorescence plate reader (Labsystems, Helsinki, Finland). Three replicates were analyzed for each time point (24, 48, 72, 96 and 120 h). The dependent T-Test was used to assess statistical significance (cutoff p ≤ 0.05).

### Endocytosis assays and microscopy (TEM, STEM, HRTEM, EELS)

Isolated magnetosomes were deposited on formvar-covered copper grids and analyzed using a FEI Morgagni transmission electron microscope operating at 80 kV (FEI Company, Hillsboro, OR, USA) to verify the presence of the magnetosome membrane after the purification process.

Approximately 10^6^ HeLa cells were seeded in 6-well round-bottom plates and 740 μg/ml of magnetosomes were add to the culture after 12 h of incubation. After 24 h of incubation, the monolayer was extensively washed to remove unbound magnetosomes. Cells were kept in DMEM with 10% FBS at 37°C in 5% CO_2_ and harvested by addition of trypsin/EDTA after periods of 24, 48, 72 and 120 h. The collected cells were washed with PBS buffer, fixed in 2.5% glutaraldehyde in 0.1 M sodium cacodylate buffer containing 3.7% sucrose and 5 mM calcium chloride for 1 h at room temperature, washed three times in the same buffer, post-fixed with 1% buffered osmium tetroxide, dehydrated in acetone series and embedded in Polybed 812 epoxy resin. Ultrathin sections were prepared using an EM UC6 microtome (Leica Microsystems), recovered on 400-mesh copper grids, stained with uranyl acetate and lead citrate and observed on FEI Morgagni transmission electron microscope (FEI Company, Hillsboro, OR, USA) at 80 kV. Magnetosome crystal size ((length + width) / 2) and shape factor (width/length ratio) were determined using iTEM software (Olympus Soft Imaging Solutions). Statistical variance analyses were performed using GraphPad InStat version 3.0.

Unstained ultrathin sections were examined by conventional transmission electron microscopy (TEM), scanning transmission electron microscopy in the high angle annular dark-field (STEM-HAADF) mode, and high-resolution transmission electron microscopy (HRTEM). For HRTEM analysis, a FEI TECNAI G2 TF20 (FEI Company, Netherlands) TEM operated at 200 kV was used. For elemental identification, spectra and mapping, a JEOL 2100F (JEOL Ltd.), operating at 200 kV in STEM-HAADF mode, with a beam spot size of 0.2 nm, and a Tridiem spectrometer (Gatan Inc., Pleasanton, CA, USA) for electron energy loss spectroscopy (EELS) were used. A spatial drift corrector was used during high-resolution elemental mapping acquisition with a dwell time of 2 sec. For the images and EELS map analysis, the Digital Micrograph software (Gatan Inc., Pleasanton, CA, USA) and the JEMS software [15] were used.

## Results and discussion

**Fig 1:**
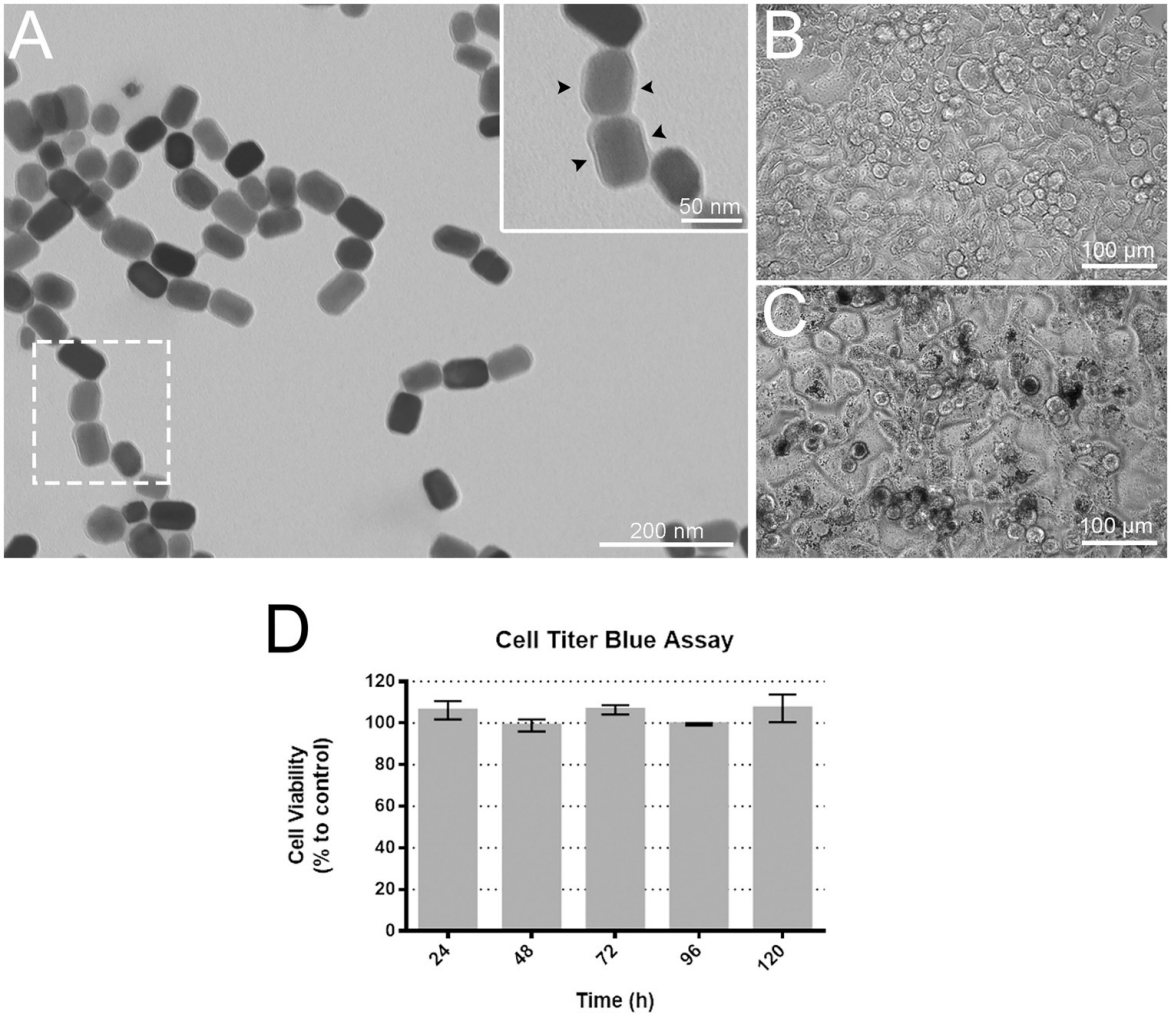
TEM image of purified magnetosomes and phase contrast light microscopy images of HeLa cell culture. A) Purified magnetosomes with surrounding membrane (inset shows the magnetosome membrane in detail; black arrowheads). B) Phase contrast image of HeLa culture in which magnetite magnetosomes were not added (control). C) Phase contrast image of HeLa culture incubated with magnetosomes purified from *Magnetovibrio blakemorei* strain MV-1. D) Cell survival rates of HeLa cells after incubation with magnetite magnetosomes. The viability of the cells was determined using the Cell titer Blue cytotoxicity assay up to 120 hours after contact with magnetosomes. No toxic effect was detected and a slight growth of cells was observed when compared to control (percentages above 100%).

TEM observation of purified magnetite magnetosomes from *M*. *blakemorei* strain MV-1 showed the presence of the magnetosome membrane surrounding each crystal (Fig 1A) confirming the homogeneity and potential reproducibility of the sample. After the endocytosis assay, phase contrast images of HeLa cells in cultures showed confluent growth on the glass surface on both control (not incubated with magnetosomes) and treated cultures even after 120 h of incubation with purified magnetosomes (Fig 1B and 1C). Treated cultures displayed dark areas corresponding to aggregates of magnetosomes; even in these areas, no inhibition of growth was detected. The cytotoxicity assay showed that magnetosomes did not have any toxic effect on cells in all incubation times analyzed (Fig 1D). Magnetosome cytotoxicity have already been evaluated against a few cell lines. In these studies, H22 (mouse hepatocarcinoma cells), HL60 (human acute promyelocytic leukemia cells), EMT-6 (mouse mammary cancer cells) and ARPE-19 cells (human retinal pigment epithelium cells) were treated with 9 μg/ml of magnetite magnetosomes for the three first cell lineages and 10, 50 and 100 μg/ml for ARPE-19 cells and no toxic effect was observed [11,12]. Qi et al. (2016) showed that when the same amounts of synthetic magnetic particles were added to ARPE-19 cells (10, 50 and 100 μg/ml), their viability decreased significantly after incubation [12]. This study also reported that cell viability rates were directly correlated to the concentration of the synthetic nanoparticles as well as the incubation period. Similar results were obtained for 661W cells (mouse transformed cell line) [12]. Therefore, cell death was directly related to an increase in the concentration of synthetic magnetite nanoparticles incubated with cell cultures. On the other hand, the use of the same concentrations of magnetosomes promoted the growth of ARPE-19 cells [12]. For 661W cells, 10 μg/ml magnetosomes promoted cell growth during all incubation periods used in the study (24, 48 and 72 h) [12]. Therefore, our findings are consistent with these previous studies, indicating high biocompatibility between magnetosomes and mammalian cells even when high concentrations of magnetosomes were used. In addition to the non-deleterious effect on cells, the presence of a biological membrane involving the magnetosome also make these magnetic nanoparticles excellent tools in nanomedicine, because of the possibility of associating molecules to proteins embedded on this outer phospholipidic bilayer.

**Fig 2:**
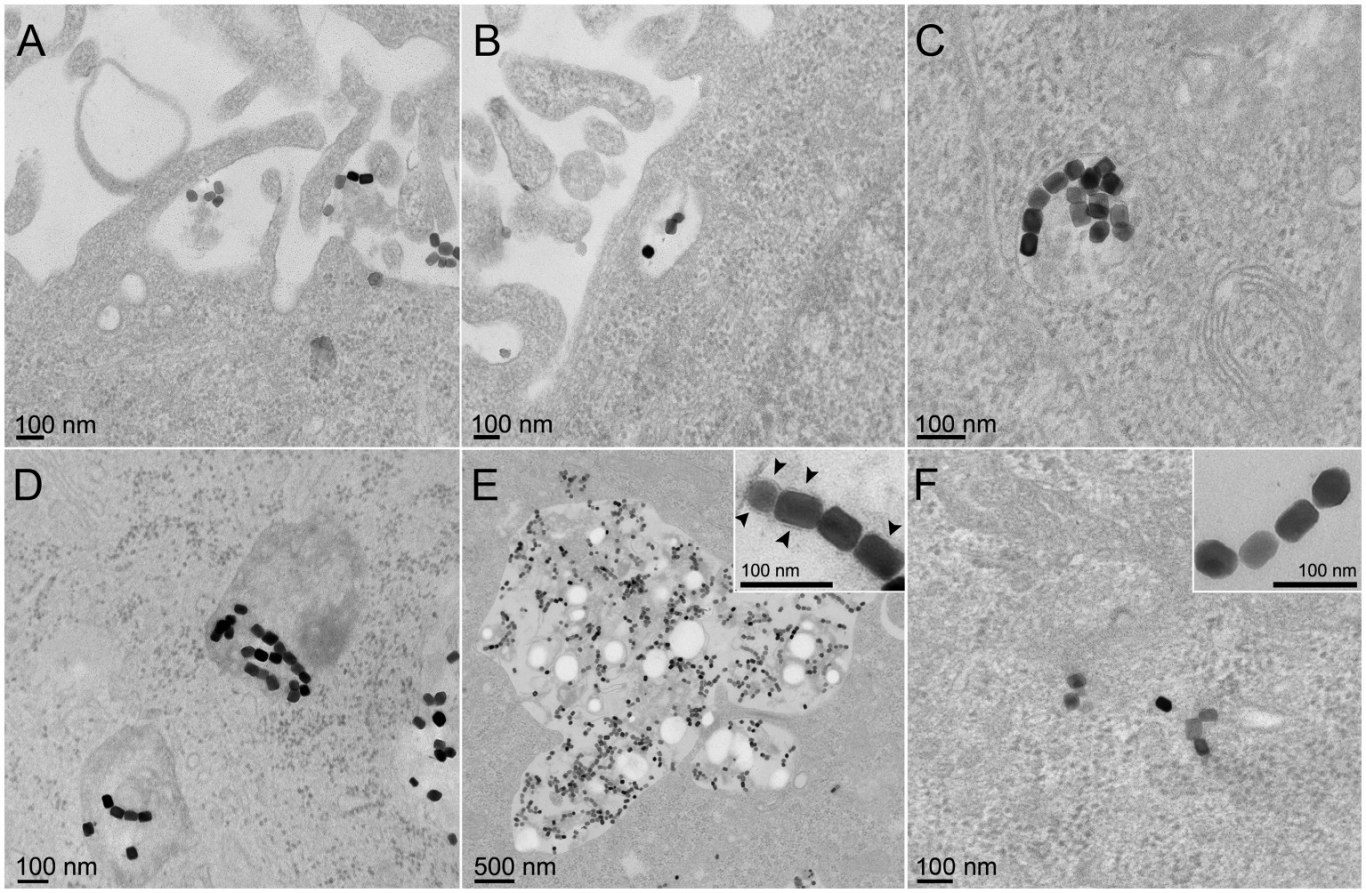
TEM images of stained ultrathin sections of HeLa cells showing internalization of magnetite magnetosomes purified from *M*. *blakemorei* strain MV-1. A) Magnetosomes at the surface of cells showing the first steps in endocytosis. B; C; D; E) Endocytic vacuoles in the cell cytoplasm containing magnetosomes, some of them smaller without lysosomes, close to cell membrane (B), deep inside cell cytoplasm (C), and (D), or in large endocytic vacuoles with magnetosomes and lysosomes (E), the inset shows magnetosomes in endocytic vesicles in detail (arrowheads indicate the magnetosome membrane). F) Magnetosomes in the cytoplasm of the cell, the inset shows magnetosomes from this region in detail, showing the absence of the magnetosome membrane.

Ultrathin sections of HeLa cells after incubation with magnetosomes showed a large number of internalized particles in endosome vesicles apparently in different stages of endocytosis (Fig 2A–2E), confirming that magnetosomes crystals followed the endocytic pathway observed in other cell lineages [10,12]. Interestingly, in this study, some magnetosomes were also detected in the cytoplasm (Fig 2F). This observation has not been reported before and suggests that magnetosomes might have been taken up by the cell by different mechanisms. TEM showed that the membrane of magnetosomes was still present in magnetosomes located within endosomes (Fig 2E inset; arrowheads), but absent from those found in the cytoplasm outside endocytic vesicles (Fig 2F inset). The absence of the enveloping membrane on magnetic nanocrystals observed in the cytoplasm suggests that the membrane of these magnetosomes was removed from the crystal after endocytosis and the magnetic nanocrystal was transferred to the cytoplasm. The endosomal scape could be promoted by unknown endosomolytic agents present in the magnetosome. Magnetosomes, as well as their associated proteins, have never been tested for their endosomolytic potential, but this should probably opportune because of the benefits to the development of novel drug-delivery systems [16]. Another possibility to explain the absence of membrane in magnetic nanoparticles within the cytoplasm is that some magnetosomes could have lost the membrane during the purification process and were guided to a different site within the cell. Eukaryotic cells promote endocytosis by different means that are dependent on the size of the particle(s) and the chemical properties of molecules on the structures that are being internalized [17]. Because magnetosomes found “freely” in the cytoplasm were relatively low in number and the aggregates of particles, in this case, were at the nanometer scale, it is possible that a different mechanism was used to internalize single or a very small aggregates of magnetosomes. Magnetosomes in endocytic vesicles were probably engulfed by cells as aggregates of particles at the micrometer scale. In addition to this, the absence of the magnetosome membrane might have modified the chemical properties of the magnetosomes possibly also leading to this different mechanism of endocytosis, as previously mentioned.

No obvious alteration in magnetosomes’ magnetite crystal morphology was observed in either case, magnetosomes in endocytic vesicles or within the cytoplasm of the cells. Size and shape factor of magnetite crystals in freshly purified magnetosomes and those in endocytic vesicles or the cytoplasm after internalization by HeLa cells showed no significant differences (S1 Fig). HRTEM images of internalized magnetosomes (S2A and S2B Fig) were consistent with the mineral magnetite (Fe_3_O_4_). Variations in the crystalline structure were not detected even in the nanocrystals without the membrane in the cytoplasm, indicating that magnetite magnetosomes were not promptly dissolved. Therefore, toxic effects would not be generated due to the dissolution of the crystals and the significant release of great amounts of soluble iron in the cell. From the observations above we can consider that both magnetosomes with and without a surrounding membrane are stable inside cells for a significantly long period, allowing us to glimpse new applications for their use such as localization of specific targets, controlled drug delivery, hyperthermia assays, enhancement of medical imaging, among others.

**Fig 3:**
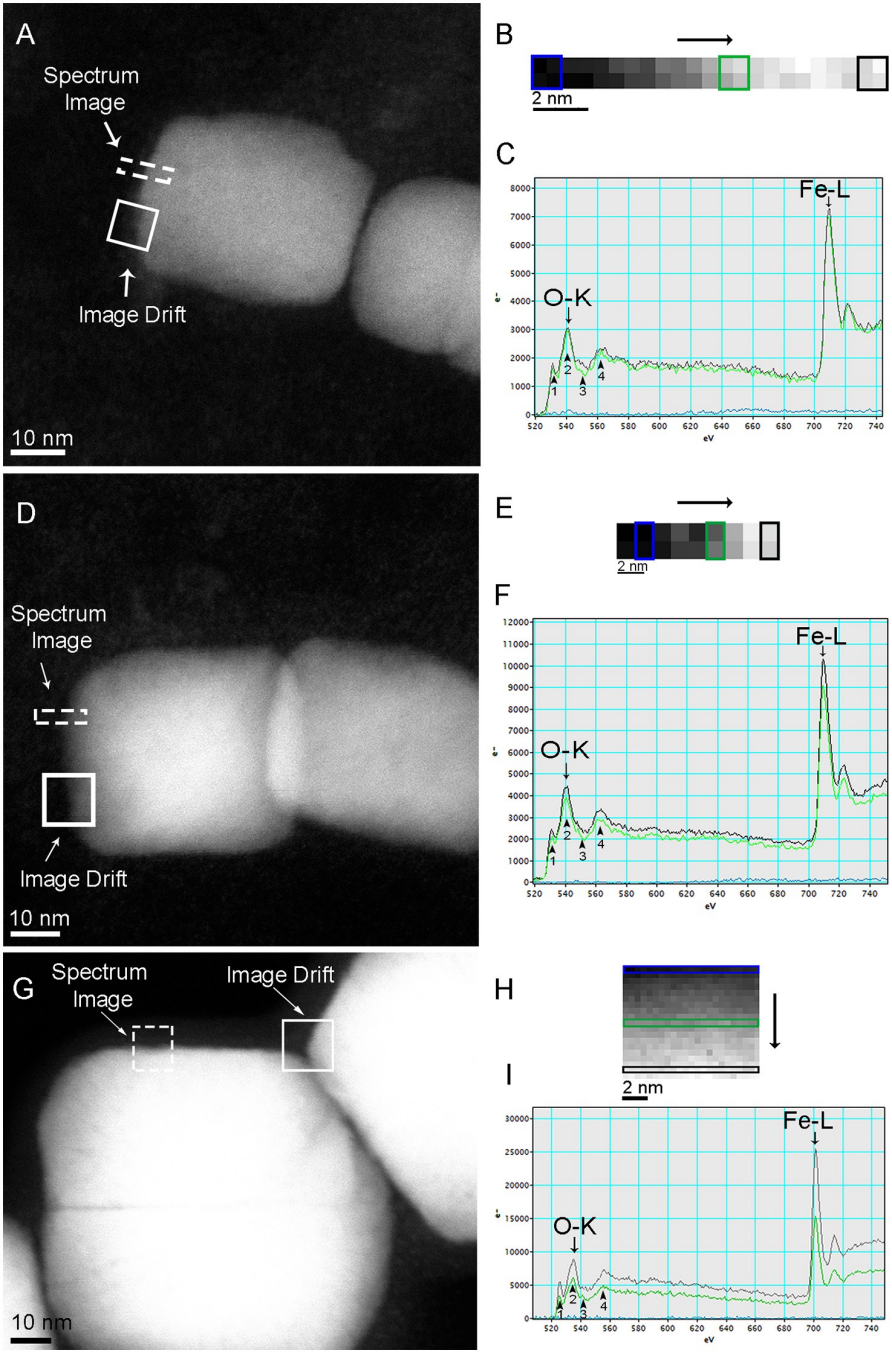
STEM-EELS analysis of magnetosomes’ magnetite crystals in endosome (A-C), directly within the cytoplasm of a HeLa cell (D-F), and freshly purified magnetosome (control; G-I). A) STEM-HAADF image of magnetosomes showing the areas used for analysis (dashed rectangle) and drift correction (solid square). B) Spectrum map of the selected area in (A), showing the scanning direction (black arrow) and pixels used to obtain the EELS spectra outside (blue square), next the border (green square) and on bulk crystal (black square). C) EELS spectra outside crystal (blue line), near the crystal border (green line), and on the bulk crystal (black line) showing characteristic edges (arrows) for O (540 eV) and Fe (710 eV). D) STEM-HAADF image of magnetosome crystal within the cytoplasm showing the area selected for EELS analysis (dashed rectangle) and drift correction (solid square). E) Spectrum map of the selected area in (D), showing the scanning direction (black arrow) and pixels used to obtain the EELS spectra outside (blue rectangle), next the border (green rectangle) and on the bulk crystal (black rectangle) F) EELS spectra outside crystal (blue line), near the crystal border (green line), and on the bulk crystal (black line) showing characteristic edges (arrows) for O (540 eV) and Fe (710 eV). G) STEM-HAADF image of purified magnetosome showing the area selected for EELS analysis (dashed square) and drift correction (solid square). Faint dark straight lines inside crystals images could correspond to defects of the original nanocrystals. H) Spectrum map of the selected area in (G), showing pixels used to obtain the EELS spectra outside (blue rectangle), next the border (green rectangle) and on the bulk crystal (black rectangle). I) EELS spectra outside crystal (blue line), near the crystal border (green line), and on the bulk crystal (black line) showing characteristic edges (arrows) for O (540 eV) and Fe (710 eV). Black arrowheads in (C, F and I) show the specific of O-K edge peaks for iron oxides; in these cases similar to magnetite (see Colliex et al., 1991 [18]). Note that the bulk and the border O-K spectra are similar in all samples.

**Fig 4:**
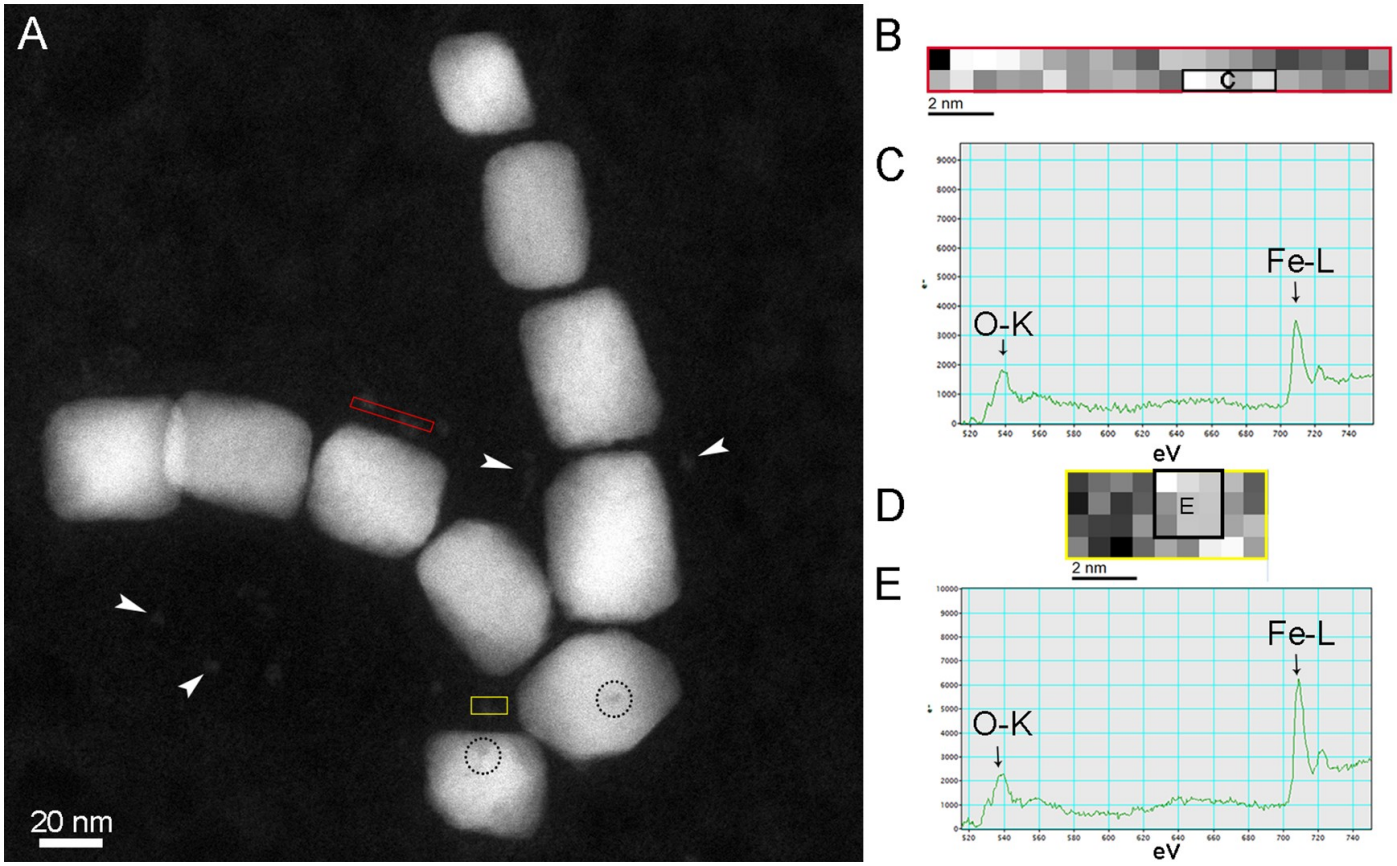
STEM-EELS Images and spectrum map of fine crystalline structures near magnetosome crystals. A) STEM-HAADF image of magnetosomes in the cytoplasm of HeLa cell showing the areas selected for EELS spectrum maps (red and yellow rectangles). White arrowheads in (A) show other equivalent structures and black dashed circles indicate defects/damage in the crystal. B) Gray value spectrum map of the selected area in red rectangle (A); pixels used to obtain the EELS spectra (C) are represented in the black rectangle. C) EELS spectra of the region indicated by the black rectangle in (B) showing characteristic edges (arrows) for O (540 eV) and Fe (710 eV). D) Grey value spectrum map of the selected area in yellow rectangle (A); pixels used to obtain the EELS spectra in (E) are represented in the black rectangle. E) EELS spectra of the region indicated by the black rectangle in (D) showing characteristic edges (arrows) for O (540 eV) and Fe (710 eV). Note that the specific of O-K and Fe-L edge peaks for iron oxides, which were observed in Fig 3C, 3F and 3I, are not detected on (C) nor (E).

EELS mapping and spectra of the energy loss near edge structure (ELNES) of oxygen (O-K edge around 540 eV) and iron (Fe-L edge around 710 eV) were done in order to compare iron oxidation states on the bulk, near the border and outside the magnetosome in endosome (Fig 3A and 3B), cytoplasm (Fig 3D and 3E) and control (Fig 3G and 3H). According to EELS spectra, magnetosomes inside (Fig 3C) and outside endocytic vesicles (Fig 3F) presented the same configuration as the purified magnetosomes (control; Fig 3I), which is consistent with magnetite [18,19]. No change in the oxidation state was observed even at the border of the mineral (Fig 3C and 3F black and green lines, respectively), suggesting that these nanoparticles are not undergoing chemical modifications by the cell after 120 h of internalization. EELS mapping near magnetosomes did not show any significant free iron concentration in this area (Fig 3C and 3F; blue line on both images), confirming that these magnetosomes were not submitted to a rapid process of degradation/dissolution. On the other hand, smaller structures of approximately 5 nm were observed near the magnetosomes in endosomes and within the cytoplasm (Fig 4A and S2C Fig). HRTEM shows a lattice plane in these structures with only one detected interplanar distance (2.6 Å), which cannot be used to identify the iron oxide (S2D Fig). STEM-EELS revealed the presence of iron oxide at O-K and Fe-L_2,3_ edges, but oxygen peaks did not present enough resolution to allow the specific identification of the iron oxide (Fig 4B–4E). In these cases, identification was not possible because of the low signal provided by the analysis of this fine structure considering the high noise caused by the background. In addition to these fine structures, low contrast areas were unusually observed in internalized magnetosomes (Fig 4A; dashed circles); which also suggest minor defects/damage in the crystal that would not be detected based on size and shape. In ultrathin sections, where the magnetosomes were inside endosomes, these structures might represent a minor degradation of magnetosomes, although magnetosome crystal analysis showed that there was no alteration of crystal structure nor difference in morphology and size of magnetosomes when compared to control. Dissolution-reprecipitation processes have been described during the thermal conversion of maghemite to hematite in aqueous solutions [20] and could be related to this structure, indicating the first evidence of magnetosome degradation within a mammalian cell.

Degradation of the magnetite crystal of magnetosomes by human cells is one of the most important queries for applying these nanoparticles as tools in Biomedicine. Few studies were conducted to evaluate magnetosome degradation *in vitro* using human model cell lines. The potential of magnetosome degradation by mammalian enzymes was observed in *in vitro* experiments simulating lysosomal activity with proteases [9]. However, in this approach magnetosomes were incubated for 28 days in crude protease from the bovine pancreas in a buffer solution, which might have a stronger effect on mineral degradation than that obtained by the actual metabolism of cells.

## 4. Conclusions

In this study, we evaluated the cytotoxicity and possible degradation of magnetosomes using HeLa cells as a model lineage. Our results showed that magnetosomes are biocompatible and are not oxidized or suffer morphological changes after internalization by cells. In contrast, studies using synthetic magnetic particles showed toxic effects over cells after 24 h of incubation [12]. The presence of fine crystalline iron-containing structures of approximately 5 nm near internalized magnetosomes might be an indication of a slow degradation or the detachment of small parts of the nanoparticles due to original defects (see Fig 3G). If true, this process would be a great advantage for the use of magnetite magnetosomes in many medical and biotechnological applications because it would guarantee nontoxic effects on the cell due to the derisory release of low concentrations of soluble iron over a long period. Moreover, the presence of a biological membrane in isolated magnetosomes and after some period in endocytic vesicles enlarges the range of possible applications of magnetosomes in the bioengineering area. Overall, this suggests that magnetite magnetosomes remain as stable structures in biological systems for long periods, which is also desirable for prolonged treatments.

For an appropriate, accurate comparison, a long-term *in vitro* study using high-resolution microscopy and micro/nano-analysis techniques should be performed. We suggest that studies about magnetosome degradation in different cell lines should be focussed on the analysis of the oxidation of magnetosomes magnetite crystals particularly at regions near the crystal borders by scanning transmission electron microscopy- electron energy loss spectroscopy approach as described here. Hopefully, this approach will contribute to the understanding of magnetosome degradation/persistence in biological systems. The comparison among different iron oxide nanoparticles degradation in model cell lines is necessary to acquire precise and standardized information, which are relevant to the development of new and save approaches in Nanotechnology and Biomedicine.

## Supporting information

S1 Fig Mean (A) size and (B) shape factor of magnetosomes in freshly purified (control), in endosomes and cytoplasm after HeLa cells internalization. No statically significant differences were observed among samples.
(TIF)

S2 Fig A) STEM-HAADF image of magnetosomes inside endosome; dashed square shows the crystal imaged by HRTEM (B). B) HRTEM image, and FFT (inset) of the magnetosome selected in the square of image (A), showing the crystalline structure of the prismatic magnetite crystal elongated in 111 direction in [0, –1, 1] zone axis. C) STEM-HAADF image of magnetosomes inside endosome, showing crystalline structures near the magnetosome (white square) displayed with greater exposure on the inset. D) Higher magnification of the region indicated by the square in (C) showing a crystalline structure near the magnetosome; inset shows FFT corresponding to the white square area, with a plane lattice distance of +- about 2.6 Å.
(TIF)
