# Supplementary figures and images for: Uptake and persistence of bacterial magnetite magnetosomes in a mammalian cell line: Implications for medical and biotechnological applications
Source: PLoS One. 2019 Apr 23;14(4):e0215657. doi: 10.1371/journal.pone.0215657 (PMC6478323; doi:10.1371/journal.pone.0215657)

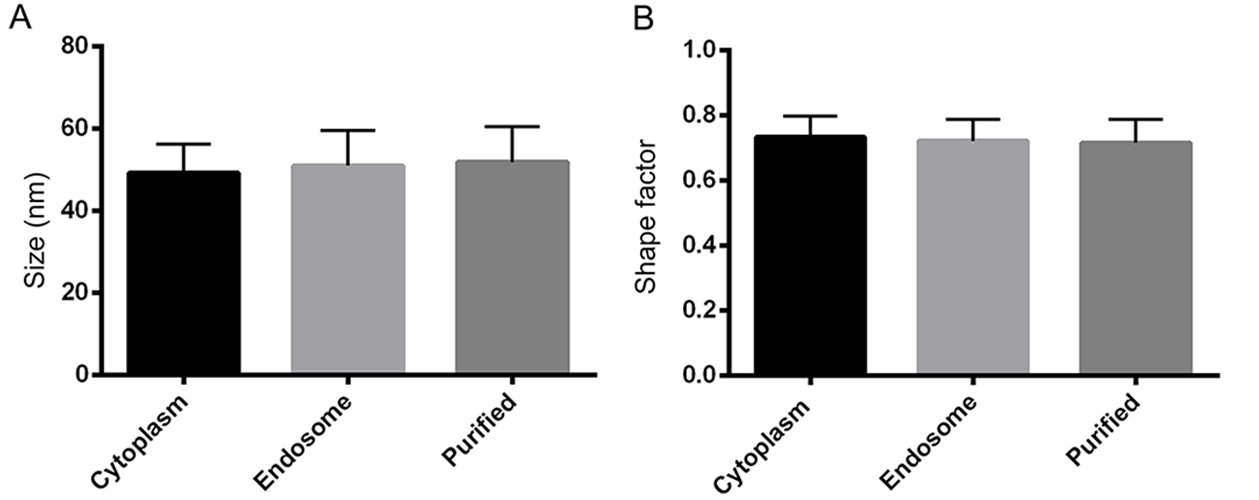

Supplement: S1 Fig — No statically significant differences were observed among samples. (TIF) [file pone.0215657.s001.tif]

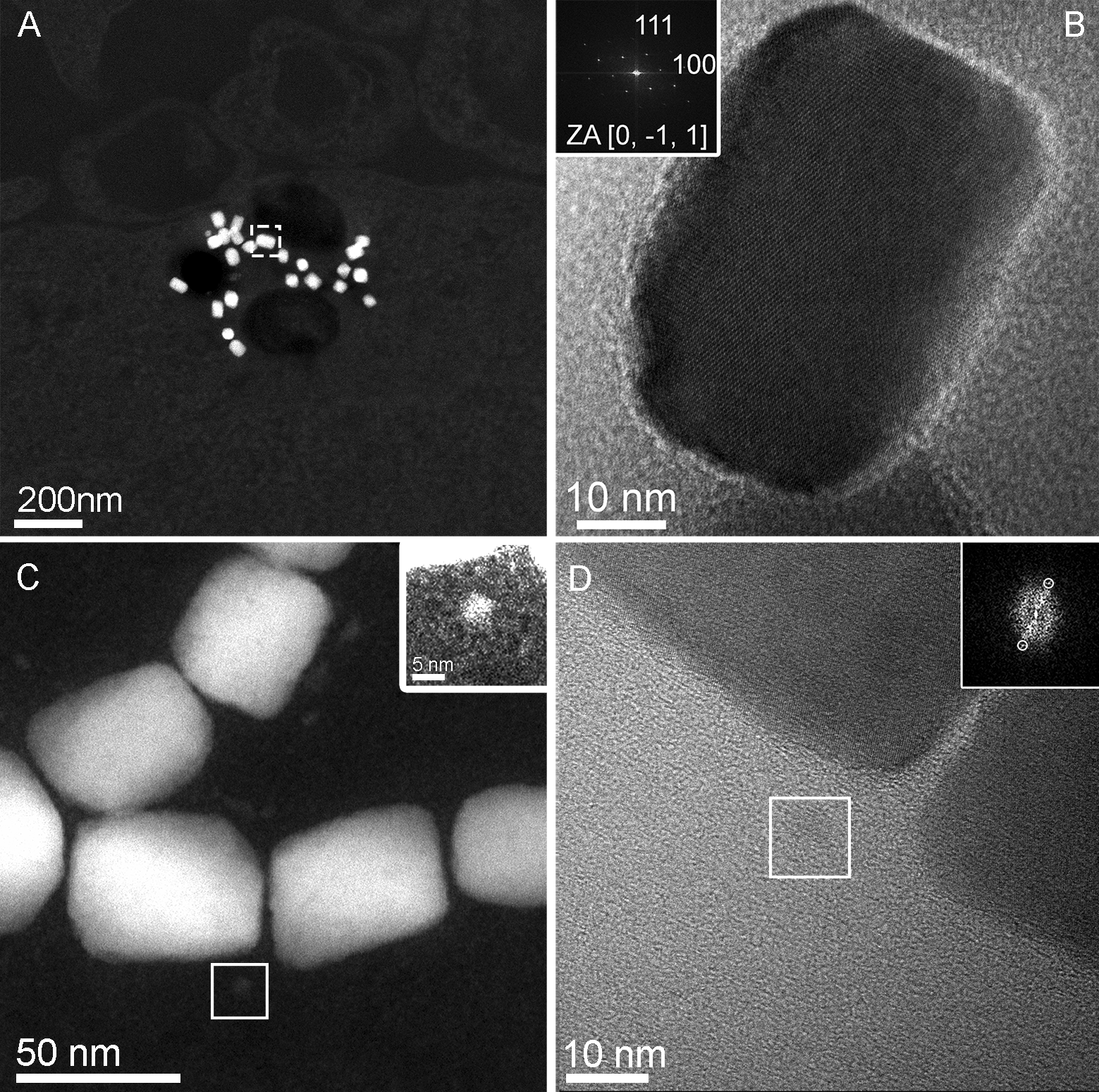

Supplement: S2 Fig — A) STEM-HAADF image of magnetosomes inside endosome; dashed square shows the crystal imaged by HRTEM (B). B) HRTEM image, and FFT (inset) of the magnetosome selected in the square of image (A), showing the crystalline structure of the prismatic magnetite crystal elongated in 111 direction in [0, –1, 1] zone axis. C) STEM-HAADF image of magnetosomes inside endosome, showing crystalline structures near the magnetosome (white square) displayed with greater exposure on the inset. D) Higher magnification of the region indicated by the square in (C) showing a crystalline structure near the magnetosome; inset shows FFT corresponding to the white square area, with a plane lattice distance of +- about 2.6 Å. (TIF) [file pone.0215657.s002.tif]
